# Supplementary material for: Experimental evidence for core-Merge in the vocal communication system of a wild passerine
Source: Nat Commun. 2022 Sep 24;13:5605. doi: 10.1038/s41467-022-33360-3 (PMC9509327; doi:10.1038/s41467-022-33360-3)
Supplement: Supplementary file 1 — Supplementary Information [file 41467_2022_33360_MOESM1_ESM.pdf]

SUPPLEMENTARY INFORMATION

Experimental evidence for core-Merge in the vocal communication system of a wild passerine

Authors

Toshitaka N. Suzuki[1,2]*, Yui K. Matsumoto[2,3]

Affiliations

[1]The Hakubi Center for Advanced Research, Kyoto University, Yoshida-honmachi, Sakyo-ku, Kyoto 606-8501, Japan.

[2]Department of Zoology, Graduate School of Science, Kyoto University, Kitashirakawa-oiwake-cho, Sakyo-ku, Kyoto 606-8502, Japan.

[3]Department of Information Medicine, National Institute of Neuroscience, National Center of Neurology and Psychiatry, 4-1-1, Ogawahigashi-cho, Kodaira, Tokyo 187-8502, Japan.

*Corresponding author, Email: toshi.n.suzuki@gmail.com

**Predator approach**				
Contrast	Estimate	SE	*Z*	*P*
1A-R vs 1R-A	4.10	0.80	5.10	< 0.0001
1A-R vs 2A-R	5.05	0.92	5.50	< 0.0001
1A-R vs 2R-A	3.76	0.77	4.90	< 0.0001
1R-A vs 2A-R	0.95	0.81	1.16	0.2938
1R-A vs 2R-A	-0.34	0.80	-0.43	0.6700
2A-R vs 2R-A	-1.29	0.85	-1.52	0.1939
**Wing flicking**				
Contrast	Estimate	SE	*Z*	*P*
1A-R vs 1R-A	5.09	1.09	4.69	< 0.0001
1A-R vs 2A-R	3.87	0.68	5.68	< 0.0001
1A-R vs 2R-A	3.65	0.66	5.54	< 0.0001
1R-A vs 2A-R	-1.22	1.15	-1.06	0.3468
1R-A vs 2R-A	-1.44	1.14	-1.26	0.3127
2A-R vs 2R-A	-0.22	0.77	-0.28	0.7772

Supplementary Table 1. Results of pairwise comparisons between treatments by using estimated marginal means (proportional data analyses). Proportion of individuals in Japanese tit flocks that approached within 2-m of the shrike specimen (upper). Proportion of individuals in Japanese tit flocks that exhibited wing flicking (lower). *P*-values are adjusted by applying false discovery rate control. Abbreviations: 1A-R: one-speaker playback of alert-recruitment sequences; 2A-R: two-speaker playback of alert calls and recruitment calls arranged in this order; 1R-A: one-speaker playback of recruitment-alert sequences; 2R-A: two-speaker playback of recruitment calls and alert calls arranged in this order. See Fig. 4.

Predator approach				
Contrast	Estimate	SE	*Z*	*P*
1A-R vs 1R-A	4.65	1.53	3.05	0.0078
1A-R vs 2A-R	4.63	1.54	3.01	0.0078
1A-R vs 2R-A	3.84	1.37	2.81	0.0098
1R-A vs 2A-R	-0.01	1.29	-0.01	0.9918
1R-A vs 2R-A	-0.81	1.22	-0.66	0.6133
2A-R vs 2R-A	-0.79	1.21	-0.66	0.6133
**Wing flicking**				
Contrast	Estimate	SE	*Z*	*P*
1A-R vs 1R-A	6.18	1.69	3.66	0.0015
1A-R vs 2A-R	4.61	1.43	3.23	0.0025
1A-R vs 2R-A	4.75	1.42	3.33	0.0025
1R-A vs 2A-R	-1.57	1.26	-1.24	0.3157
1R-A vs 2R-A	-1.43	1.28	-1.12	0.3157
2A-R vs 2R-A	0.14	0.97	0.14	0.8879

Supplementary Table 2. Results of pairwise comparisons between treatments by using estimated marginal means (binary data analyses). Whether or not at least one Japanese tit approached within 2-m of the shrike specimen (upper). Whether or not at least one Japanese tit exhibited wing flicking (lower). *P*-values are adjusted by applying false discovery rate control. Abbreviations: 1A-R: one-speaker playback of alert-recruitment sequences; 2A-R: two-speaker playback of alert calls and recruitment calls arranged in this order; 1R-A: one-speaker playback of recruitment-alert sequences; 2R-A: two-speaker playback of recruitment calls and alert calls arranged in this order. See Supplementary Fig. 1.

Block	Individual identity of source individuals used for each call set		Number of source individuals
	Alert call	Recruitment call	
No. 1	Bird no. 1	Bird no. 1	One
No. 2	Bird no. 2	Bird no. 17	Two
No. 3	Bird no. 3	Bird no. 3	One
No. 4	Bird no. 4	Bird no. 18	Two
No. 5	Bird no. 5	Bird no. 5	One
No. 6	Bird no. 6	Bird no. 19	Two
No. 7	Bird no. 7	Bird no. 7	One
No. 8	Bird no. 8	Bird no. 20	Two
No. 9	Bird no. 9	Bird no. 9	One
No. 10	Bird no. 10	Bird no. 21	Two
No. 11	Bird no. 11	Bird no. 11	One
No. 12	Bird no. 12	Bird no. 22	Two
No. 13	Bird no. 13	Bird no. 13	One
No. 14	Bird no. 14	Bird no. 23	Two
No. 15	Bird no. 15	Bird no. 15	One
No. 16	Bird no. 16	Bird no. 24	Two

Supplementary Table 3. Preparation for playback stimuli. Each block contains four playback treatments (i.e., one-speaker playback of alert-recruitment sequences, two-speaker playback of alert calls and recruitment calls arranged in this order, one-speaker playback of recruitment-alert sequences, and two-speaker playback of recruitment calls and alert calls arranged in this order) created from either a unique set of alert and recruitment calls from the same bird (n = 8 source individuals; n = 8 alert calls, n = 8 recruitment calls) or from two different birds (n = 16 source individuals; n = 8 alert calls, n = 8 recruitment calls).

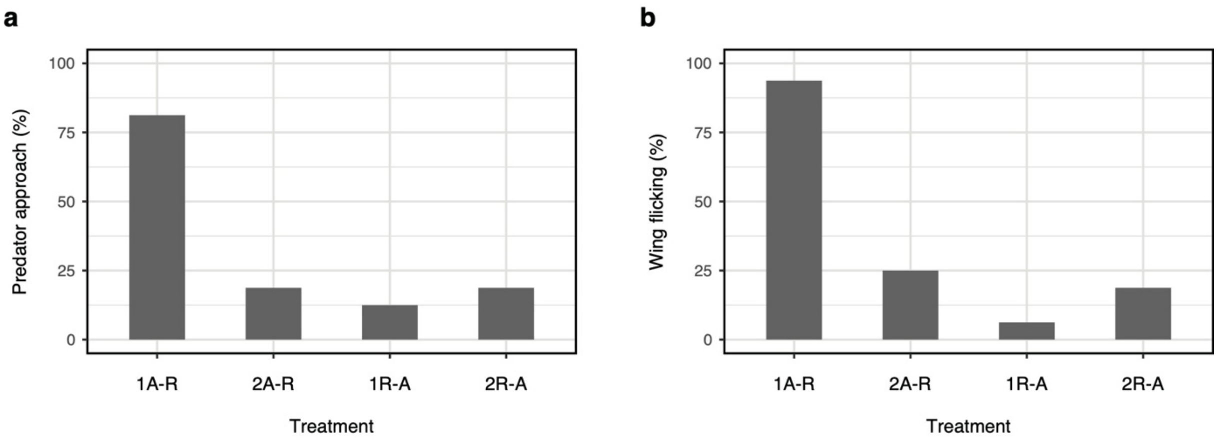

Supplementary Figure 1. Predator mobbing by Japanese tits during call playbacks. (**a**) Percentage of trials in which at least one Japanese tit approached within 2-m of the shrike specimen. Treatment (generalized linear mixed model: $\chi^2 = 27.42$, $df = 3$, $P < 0.0001$) and flock size ($\chi^2 = 7.94$, $df = 1$, $P = 0.0048$) had significant effects on receivers' responses, while no statistical significance was found for number of source individuals used for creating playback stimuli (one vs two source individuals: $\chi^2 = 0.13$, $df = 1$, $P = 0.7189$). (**b**) Percentage of trials in which at least one Japanese tit exhibited wing flicking displays. Treatment ($\chi^2 = 37.71$, $df = 3$, $P < 0.0001$) showed statistical significance, while no significant effects were found for flock size ($\chi^2 = 1.56$, $df = 1$, $P = 0.2121$) and number of source individuals ($\chi^2 = 0.01$, $df = 1$, $P = 0.904$). Statistical significance was calculated using two-sided log-likelihood ratio tests. Sample size: $n = 16$ trials for each treatment, resulting in $n = 64$ trials across all four treatments. Abbreviations: 1A-R: one-speaker playback of alert-recruitment sequences; 2A-R: two-speaker playback of alert calls and recruitment calls arranged in this order; 1R-A: one-speaker playback of recruitment-alert sequences; 2R-A: two-speaker playback of recruitment calls and alert calls arranged in this order. See Supplementary Table 2 for details of statistical analyses. Source data are provided as a Source Data file.
