# Supplementary material for: Experimental evidence for core-Merge in the vocal communication system of a wild passerine
Source: Nat Commun. 2022 Sep 24;13:5605. doi: 10.1038/s41467-022-33360-3 (PMC9509327; doi:10.1038/s41467-022-33360-3)
Supplement: Supplementary file 3 — Description of Additional Supplementary Files [file 41467_2022_33360_MOESM3_ESM.pdf]

Description of Additional Supplementary Files

File Name: Supplementary Movie 1
Description: **Mobbing of a predator by Japanese tits.** Individuals approach a shrike specimen and exhibit wing flicking displays during one-speaker playback of alert-recruitment call sequences.
