# Supplementary material for: Experimental evidence for core-Merge in the vocal communication system of a wild passerine
Source: Nat Commun. 2022 Sep 24;13:5605. doi: 10.1038/s41467-022-33360-3 (PMC9509327; doi:10.1038/s41467-022-33360-3)
Supplement: Supplementary file 5 — Reporting Summary [file 41467_2022_33360_MOESM5_ESM.pdf]

Corresponding author(s): Toshitaka N. Suzuki

Last updated by author(s): Sep 4, 2022

Reporting Summary

Nature Portfolio wishes to improve the reproducibility of the work that we publish. This form provides structure for consistency and transparency in reporting. For further information on Nature Portfolio policies, see our Editorial Policies and the Editorial Policy Checklist.

Statistics

For all statistical analyses, confirm that the following items are present in the figure legend, table legend, main text, or Methods section.

n/a	Confirmed	
☐	☒	The exact sample size (*n*) for each experimental group/condition, given as a discrete number and unit of measurement
☐	☒	A statement on whether measurements were taken from distinct samples or whether the same sample was measured repeatedly
☐	☒	The statistical test(s) used AND whether they are one- or two-sided<br>*Only common tests should be described solely by name; describe more complex techniques in the Methods section.*
☐	☒	A description of all covariates tested
☐	☒	A description of any assumptions or corrections, such as tests of normality and adjustment for multiple comparisons
☐	☒	A full description of the statistical parameters including central tendency (e.g. means) or other basic estimates (e.g. regression coefficient) AND variation (e.g. standard deviation) or associated estimates of uncertainty (e.g. confidence intervals)
☐	☒	For null hypothesis testing, the test statistic (e.g. *F*, *t*, *r*) with confidence intervals, effect sizes, degrees of freedom and *P* value noted<br>*Give P values as exact values whenever suitable.*
☒	☐	For Bayesian analysis, information on the choice of priors and Markov chain Monte Carlo settings
☐	☒	For hierarchical and complex designs, identification of the appropriate level for tests and full reporting of outcomes
☐	☒	Estimates of effect sizes (e.g. Cohen's *d*, Pearson's *r*), indicating how they were calculated

Our web collection on statistics for biologists contains articles on many of the points above.

Software and code

Policy information about availability of computer code

Data collection	No software was used in data collection.
Data analysis	Statistical analyses were conducted in R version 4.2.1 using the icc function in the package ‘irr’ (version 0.84.1), glmer function in the package ‘lme4’ (version 1.1-30), cbind function in the package ‘base’ (version 4.2.1), anova function in the package ‘base’ (version 4.2.1) and emmeans function in the package ‘emmeans’ (version 1.7.5). Playback files were created by using Audacity version 2.1.3. R codes used in the analysis are available in Figshare (https://doi.org/10.6084/m9.figshare.18007046).

For manuscripts utilizing custom algorithms or software that are central to the research but not yet described in published literature, software must be made available to editors and reviewers. We strongly encourage code deposition in a community repository (e.g. GitHub). See the Nature Portfolio guidelines for submitting code & software for further information.

Data

Policy information about availability of data

All manuscripts must include a data availability statement. This statement should provide the following information, where applicable:

- Accession codes, unique identifiers, or web links for publicly available datasets
- A description of any restrictions on data availability
- For clinical datasets or third party data, please ensure that the statement adheres to our policy

All data used in the analysis are available in Figshare (https://doi.org/10.6084/m9.figshare.18007046).

Field-specific reporting

Please select the one below that is the best fit for your research. If you are not sure, read the appropriate sections before making your selection.

☐ Life sciences ☐ Behavioural & social sciences ☒ Ecological, evolutionary & environmental sciences

For a reference copy of the document with all sections, see nature.com/documents/nr-reporting-summary-flat.pdf

Ecological, evolutionary & environmental sciences study design

All studies must disclose on these points even when the disclosure is negative.

Study description

We tested whether Japanese tits have evolved core-Merge to recognize two temporally-linked calls produced by a single individual (alert-recruitment call sequence) as a single unit. We designed experiments with four playback stimuli differing in temporal and/or spatial linkages of alert and recruitment calls.

We used an independent measures design to test the response of 64 flocks of Japanese tits to 64 playback stimuli (n = 16 trials for each treatment). We analyzed the data by using generalized linear mixed models with a binomial error distribution and logit-link function. In the models, we treated percentages of individuals within flocks exhibiting (i) predator approach or (ii) wing flicking displays as a dependent variable. We fitted playback treatments as a fixed term, whereas flock size and the way of creating playback stimuli (whether the two call types were recorded from single individual or two individuals) as covariates. We also included identity of call combinations used for playback stimuli (n = 16 blocks) and identity of shrike specimens (n = 2) as random terms.

Research sample

We studied 64 flocks of Japanese tits in mixed deciduous-coniferous forests in Nagano and Gumma, Japan. TNS has continued field research since 2005 and thus we had the information on predators and recordings of Japanese tits' vocalizations of this population (Suzuki 2014, Suzuki et al. 2016, Suzuki et al. 2017). Although most of the birds had not been individually colour-ringed, all the experimental trials were conducted at least 400 m apart; previous observations on colour-ringed individuals showed that this distance was enough to ensure the collection of data from different individuals (Suzuki 2012).

Sampling strategy

We determined the sample size by considering the data independency and the area of study site. We conducted all experiments at different locations separated by at least 400 m apart, ensuring the collection of data from different individuals. With this method, we could collect a maximum of 64 samples within this study area. We expected that this sample size would be sufficient based on the results of our previous playback studies on the study species (Suzuki et al. 2016, Suzuki et al. 2017).

Data collection

We (TNS and YKM) conducted experiments and collected data. First, we searched for and located a flock of Japanese tits. Upon finding a flock, we fixed a taxidermic specimen of bull-headed shrike on a tree branch. Then, we also placed either one or two Bluetooth speakers on tree branches, and oriented them upwards to control for the possible influence of directionality. We set the distance between the shrike specimen and the speaker(s) at 5 m. For trials with two speakers, we set the distance between speakers at 10 m, mimicking the situation in which two birds are calling. The shrike specimen was first covered with a black cloth, and was exposed by removing the cloth just before each trial.

We began playbacks when at least two Japanese tits were present within 15 m from the shrike specimen. During 90-s of playbacks, we recorded (i) whether birds approached within 2-m of the speaker during the playback and (ii) whether they exhibited wing flicking displays. We counted the number of individuals within 15 m from the shrike during 90-s of playbacks and considered it as flock size. During trials, we sat on the ground at ca. 10 m from the shrike specimen to decrease the influence of the observers' presence on bird behaviour.

Timing and spatial scale

We conducted experiments from 26th October to 4th December 2020, in mixed deciduous-coniferous forests in Nagano and Gumma (36°17-31'N, 138°26-39'E), Japan. In this period, Japanese tits form flocks and often exhibit group mobbing behaviour against perched predators, including bull-headed shrikes.

Data exclusions

When the first bird to approach the shrike specimen was from a heterospecific species, such as a varied tit (n = 1) or a long-tailed tit (n = 1), we repeated the same experiment at a different site. This pre-established criterion allowed us to account for the possibility that the presence of heterospecific mobbers may elicit mobbing response in Japanese tits.

Reproducibility

To confirm reproducibility, we replicated n = 12 trials (3 trials for each of the four treatments) to 12 flocks of Japanese tits. All attempts to repeat the experiment were successful.

Randomization

We conducted 64 experiments for 64 flocks so that each flock was exposed to only one playback stimulus. Experiments involved four treatments of playbacks differing in temporal and/or spatial linkages of two call types. We prepared 16 blocks of playback stimuli, each of which contained all four treatments constructed from the same recording sources. The order of trials was randomized within each block (n = 16) so that responses to all four treatments were observed under largely similar conditions.

Blinding	As this experiment was based on auditory playbacks in the field, the observers could not be blind to the treatments during controlling playbacks and measuring behavioural responses. Instead, all the data were collected by two observers (TNS and YKM) and inter-observer reliability was assessed by calculating intra-class correlation coefficient (ICC). The lowest ICC was 0.998, indicating high degree of inter-observer reliability for all the behavioural measurements. We also video-recorded the responses of Japanese tits using a digital video camera. After each trial, we confirmed the exact location at which each bird made the closest approach by checking the video recording at each experimental site. Then, using a tape measure, we recorded the minimum approach distance of birds to the shrike specimen. Thus, our final data set consisted of the most reliable observations confirmed by two experimenters and video evidence.

Did the study involve field work? ☒ Yes ☐ No

Field work, collection and transport

Field conditions	We conducted experiments under calm weather conditions. We did not conduct trials under wet and windy weather conditions, since these may influence behavioural patterns of forest birds (e.g., Grubb 1975).
Location	We conducted experiments in mixed deciduous-coniferous forests in Nagano and Gumma (36°17-31'N, 138°26-39'E), Japan.
Access & import/export	We conducted experiments in national forests under the permission of the Ministry of the Environment (no. 2004245, date of issue: May 5, 2020) and the Forestry Agency of Japan (date of issue: April 1, 2020). We carefully designed experiments to minimize the influence of experiments on wild bird populations (see below). We did not collect any samples that require importing or exporting.
Disturbance	We minimized the influence of playback on wild birds by conducting only one short playback (90-s) at each site. We observed that birds always returned to normal foraging behaviour within 5 minutes after the end of playbacks.

Reporting for specific materials, systems and methods

We require information from authors about some types of materials, experimental systems and methods used in many studies. Here, indicate whether each material, system or method listed is relevant to your study. If you are not sure if a list item applies to your research, read the appropriate section before selecting a response.

Materials & experimental systems

n/a	Involved in the study	
☒	☐	Antibodies
☒	☐	Eukaryotic cell lines
☒	☐	Palaeontology and archaeology
☐	☒	Animals and other organisms
☒	☐	Human research participants
☒	☐	Clinical data
☒	☐	Dual use research of concern

Methods

n/a	Involved in the study	
☒	☐	ChIP-seq
☒	☐	Flow cytometry
☒	☐	MRI-based neuroimaging

Animals and other organisms

Policy information about studies involving animals; ARRIVE guidelines recommended for reporting animal research

Laboratory animals	This study did not involve laboratory animals.
Wild animals	We collected data from free-living flocks of Japanese tits (n = 64). We did not capture them.
Field-collected samples	This study did not involve samples collected from the field.
Ethics oversight	All protocols were approved by the ethics committee of Kyoto University (no. 202016, date of issue: April 1, 2020) and adhered to Guidelines for the Use of Animals of the Association for the Study of Animal Behaviour/Animal Behavior Society.

Note that full information on the approval of the study protocol must also be provided in the manuscript.
